# Serum interleukin-38 levels correlated with insulin resistance, liver injury and lipids in non-alcoholic fatty liver disease

**DOI:** 10.1186/s12944-022-01676-0

**Published:** 2022-08-10

**Authors:** Jun Cao, Lin Hua, Shipei Zhang, Jinping Tang, Fan Ke, Zhouhuan Wu, Guohui Xue

**Affiliations:** 1 Department of Biochemistry and Molecular Biology, School of Medicine, Jiujiang University, 17# Lufeng Road, Jiujiang, 332000 Jiangxi Province China; 2 Department of Clinical Laboratory, Jiujiang NO.1 People’s Hospital, 48# The South of Taling Road, Jiujiang, 332000 Jiangxi Province China; 3 Department of Endocrinology, Jiujiang NO.1 People’s Hospital, 48# The South of Taling Road, Jiujiang, 332000 Jiangxi Province China; 4 Department of pharmacology, School of Medicine, Jiujiang University, 17# Lufeng Road, Jiujiang, 332000 Jiangxi Province China

**Keywords:** Non-alcoholic fatty liver disease, IL-38, Biomarker, Insulin resistance, Inflammation

## Abstract

**Background:**

Insulin resistance, liver injury and dyslipidemia are reported in non-alcoholic fat liver disease (NAFLD) patients. Interleukin (IL)-38 may take part in the pathophysiology of insulin resistance. Nevertheless, the function of IL-38 in NAFLD is unknown. Herein, we determined whether serum IL-38 level might be utilised as a biochemical marker for diagnosing NAFLD.

**Methods:**

NAFLD patients and healthy participants (*n* = 91 each) were enrolled. Circulating serum IL-38 levels were detected using enzyme-linked immunosorbent assay. Other metabolic and inflammatory indices related to NAFLD were also assessed.

**Results:**

Patients with NAFLD had higher serum IL-38 levels than healthy individuals. Significantly higher serum IL-38 levels were found in patients with severe and moderate NAFLD than in patients with mild NAFLD. IL-38 showed a significant correlation with parameters of insulin resistance, inflammation, and liver enzyme in NAFLD cases. Anthropometric, insulin resistance, inflammatory parameters, lipids and frequency of NAFLD showed significant differences among the serum IL-38 level tertiles. Participants in the 2nd and 3rd tertiles of serum IL-38 levels had a greater risk of NAFLD than those in the 1st tertile. Furthermore, IL-38 ROC curve showed a high area under ROC with 0.861.

**Conclusions:**

It is possible for serum IL-38 to be a biomarker for NAFLD.

## Background

Non-alcoholic fatty liver disease (NAFLD) is a frequent chronic liver condition that has been classified as an emerging epidemic and is generally seen as part of metabolic syndrome [1]. Changes in lifestyle and diet have led to the development of NAFLD as a global public health issue [2]. Data from current epidemiological studies and the Global Burden of Disease indicate that NAFLD incidence is growing and the disease will become more dangerous, and has already affected 25% of the adult population worldwide [3–5]. Moreover, in Western countries, NAFLD is the leading cause of liver transplantation and hepatocellular cancer [6]. Liver biopsy is not practical for screening the general population as it is invasive and risky [7]. Abdominal ultrasound is the most extensively used and recognised non-invasive technique for diagnosing NAFLD. However, accurate assessment of NAFLD using abdominal ultrasound is challenging, particularly given the difficulty in discriminating between steatosis and steatohepatitis, and it does not perform well in individuals with abdominal obesity [8]. A simple, objective, reproducible, non-invasive serological biomarker that can help in the early diagnosis and identification of the severity of NAFLD is therefore urgently required.

NAFLD is closely related to phenotypic changes in the hepatocytes. Peroxisome proliferator-activated receptor gamma, a critical adipogenic transcriptional factor, promotes adipogenic transformation and lipid accumulation in the hepatocytes of NAFLD patients [9]. Some signal molecules that regulate adipogenesis, including retinoic acid, adiponectin, and chemerin, have been referred as potential diagnostic markers for NAFLD [10, 11]. Furthermore, the development of NAFLD is accelerated by inflammation, and elevated pro-inflammatory cytokines are associated with progressive pathological fibrosis and cirrhosis [12, 13]. IL-38 is a recently discovered cytokine [14]. Evidence suggests that IL-38, like its family members, may involve in homeostasis of immune cells and exert anti- or pro-inflammatory actions [15]. Several researches have demonstrated the level of IL-38 is aberrant in diabetes mellitus patients and that it can suppress inflammatory responses, reduce liver fat, and attenuate insulin resistance [16–19]. IL-38 may participate in NAFLD since its correlation with insulin resistance and hepatic inflammation caused by fat deposition in the liver. Hence, we will investigate the correlation between IL-38 and NAFLD using.

## Methods

### Participants and study design

Ninety-one NAFLD patients (NFs) and 91 healthy controls (HCs) of matched age and sex who underwent annual health examinations from 1 October 2019 to 31 September 2020 were recruited as study participants. This cohort was obtained from Jiujiang No.1 People’s Hospital. The diagnosis of the 91 NAFLD cases was ascertained using radiological criteria. All NFs followed the guidelines for prevention and treatment of NAFLD (2018, China) [20]. The exclusion criteria for the NFs were as follows: (1) weekly ethanol consumption ≥140 g for men or ≥ 70 g for women; (2) cases with drug-induced liver disease, Wilson’s disease, or cancer; (3) cases with viral hepatitis or autoimmune liver disease; (4) cases with autoimmune diseases or infections; (5) cases with obstructive sleep apnoea syndrome or polycystic ovary syndrome; (6) usage of non-steroidal anti-inflammatory drugs within the last 3 months; (7) concurrent diabetes mellitus, cholestatic liver disease, pregnancy, substantial diseases of important organs, and hypothyroidism; and (8) cases with missing data or qualified blood samples. The exclusion criteria for the HCs were as follows: (1) abnormal ultrasound imaging of the liver or abnormal liver function tests in the past; and (2) other exclusion criteria applied to the NFs. In the fasting state, a 6 mL venous blood sample was collected. The separated specimens were maintained at − 80 °C until they were analyzed. This study was approved by the ethical standards of the patient-source institution and was in compliance with the Declaration of Helsinki. The informed written consent was given by all subjects.

### Demographic, anthropometric, and laboratory evaluations

Participants’ demographic information such as age; sex; alcohol consumption; medical and medication history; and anthropometric variables; including weight and height, were collected. Counts of blood cells were obtained using the Sysmex XN2000 automatic haematology analyser. Biochemical indices were carried out by an Hitachi 7600 automatic biochemical analyzer, including alanine aminotransferase (ALT), fasting blood glucose (FBG), aspartate aminotransferase (AST), albumin (ALB), prealbumin (PA), urea, high-density lipoprotein cholesterol (HDL-C), creatinine (CREA), triglycerides (TRIG), cholesterol (CHOL), and low-density lipoprotein cholesterol (LDL-C). Serum insulin (INS), C-peptide (CpS), and IL-6 levels were detected by electrochemiluminescence immunoassay using the Cobas E602 system (Roche, Basel, Switzerland). Haemoglobin A1c (HbA1c) was measured using the MQ-6000PT HbA1c detection system with high-performance liquid chromatography. Concentration of 25 Hydroxy-vitamin D [25(OH)D] was quantified by chemiluminescent immunoassay in the MAGLUMI 4000 Plus Analyser (Snibe Co., Ltd., Shenzhen, China). C-reactive protein (CRP) was detected using the nephelometric immunoassay method on the IMMAGE 800 system. Next, for the determination of body mass index (BMI), NAFLD fibrosis score (NFS), homoeostasis model assessment of insulin resistance (HOMA-IR), and quantitative insulin sensitivity check index (QUICK), the following formulas were used: BMI = weight (kg)/height (m); NFS = ˗1.675 + 0.037 × age (years) + 0.094 × BMI (kg/m^2^) + 1.13 × Impaired fasting glucose or type 2 diabetes mellitus (yes = 1, no = 0) + 0.99 × AST/ALT ratio − 0.013 × PLT (× 10^9^/L) − 0.66 × ALB (g/dL), with impaired fasting glucose defined as FBG ≥ 110 mg/dL; HOMA-IR = fasting glucose (mmol/L) × fasting insulin (mU/L)/22.5; and QUICK = 1/[log fasting insulin (mU/L) + log fasting glucose (mg/dL)].

### NAFLD diagnosis

Upper abdominal ultrasonography was completed by two qualified radiologists. Ultrasonographic degrees of NAFLD were categorised into three types, mild, moderate, and severe, according to the Chinese standard [21].

### Quantitation of serum IL-38 concentration

An enzyme-linked immunosorbent assay (ELISA) kit (DY9110–05, R&D Systems) was used to detect the level of serum IL-38 in all participants following the manufacturer’s instructions. The assay range was 31.2–2000 pg/mL. For each sample, the concentration of IL-38 in each sample was obtained according to a standard curve constructed using the appropriate recombinant IL-38.

### Statistical analyses

Statistical analyses were completed by SPSS 23.0 software and R 4.1.2 Project for Statistical Computing. Normality was examined using the Shapiro–Wilk test. The independent *t*-test was used to compare the means of continuous variables when the data met the normal distribution; otherwise, Mann–Whitney U test was employed. χ^2^ test was applied for comparison of count data. And correlation analysis was analyzed by Spearman’s correlation. R was used to draw receiver operating characteristic (ROC) curves and performance assessment. *p* value < 0.05 was regarded as significance.

## Results

### Characteristics of the subjects

No statistical difference in subject age [32.00 (25.50,41.00) vs. 35.00 (28.00,41.50), *p* = 0.235], sex [56/35 vs. 59/32, *p* = 0.645], and serum levels of ALB [48.60 (47.50,50.65) vs. 49.20 (47.85,51.00), *p* = 0.199] was found between NFs and controls. Significant disparities in the anthropometric and laboratory data were shown in Table 1. As predicted, NF patients exhibited higher BMI than HCs (*p* < 0.05). Serum concentrations of AST, ALT, FBG, INS, CpS, HbA1c, PA, urea, CREA, TRIG, CHOL, and LDL-C were obviously higher in NFs than HCs (all *p* < 0.05). PLT and 25(OH) D were lower in NFs than in HCs (all *p* < 0.05). NFs exhibited higher levels of WBC, NEU, IL-6, and CRP than HCs (all *p* < 0.05). The two indicators of insulin resistance, HOMA-IR and QUICK, showed notable differences between the two groups. NFS, an internationally recognised biomarker for liver fibrosis degree, was also elevated in NFs.

**Table 1:** Demographic, anthropometric and laboratory variables in all subjects

Indexes	HCs(*n* = 91)	NFs(*n* = 91)	*P* value
Age(y)	32.00(25.50,41.00)	35.00(28.00,41.50)	0.235
Gender(M/F)	56/35	59/32	0.645
BMI (kg/m2)	21.67 (19.81,23.91)	25.51(24.22,27.34)	< 0.0001
ALT(U/L)	15.10(10.65,20.20)	29.80(19.55,43.30)	< 0.0001
AST(U/L)	20.30(17.90,24.35)	30.90(23.90,40.20)	< 0.0001
FBG (mmol/L)	4.64(4.435,4.815)	5.06(4.725,5.675)	< 0.0001
INS (mU/L)	6.99(5.46,8.49)	12.04(8.18,15.15)	< 0.0001
CpS	1.76(1.47,2.09)	2.69(2.32,3.31)	< 0.0001
HbA1c(%)	5.30(5.20,5.50)	5.70(5.30,5.90)	< 0.0001
HOMA-IR	1.41 (1.09,1.78)	2.73(1.88,4.07)	< 0.0001
QUICK	0.67(0.62,0.72)	0.56(0.51,0.62)	< 0.0001
WBC(×10^9^)	5.44(4.77,6.09)	6.45(5.52,7.59)	< 0.0001
NEU(×10^9^)	2.95(2.51,3.59)	3.66(2.93,4.61)	< 0.0001
PLT(×10^9^)	240.00(213.00,290.00)	221.00(182.00,255.00)	< 0.0001
ALB(g/L)	48.60(47.50,50.65)	49.20(47.85,51.00)	0.199
PA (mg/L)	243.00(213.50,264.00)	286.00(269.00,329.00)	< 0.0001
Urea (mmol/L)	4.41(3.77,4.99)	4.85(4.11,5.73)	0.010
CREA (μmol/L)	58.00(50.50,67.00)	72.00(63.00,78.00)	< 0.0001
TRIG (mmol/L)	0.91(0.73,1.24)	2.09(1.58,3.07)	< 0.0001
CHOL (mmol/L)	4.45(4.04,5.02)	5.01(4.68,5.80)	< 0.0001
HDL-C (mmol/L)	1.59(1.36,1.76)	1.24(1.06,1.42)	< 0.0001
LDL-C (mmol/L)	2.53(2.03,2.83)	2.91(2.60,3.47)	< 0.0001
IL-6	1.59(1.08,2.49)	2.42(1.55,3.52)	0.002
25(OH)D	20.30(17.35,23.55)	16.40(12.95,19.80)	< 0.0001
CRP	0.16(0.13,0.23)	0.25(0.18,0.38)	< 0.0001
NFS	−3.46(−4.07,-2.80)	−2.73(− 3.32,-2.24)	< 0.0001

*BMI* Body mass index, *ALT* Alanine aminotransferase, *AST* Aspartate aminotransferase, FBG Fasting blood glucose, *INS* Insulin, *CpS* C-peptide, *HbA1c* Hemoglobin A1c, *HOMA-IR* Homoeostasis model assessment of IR, *QUICK* Quantitative insulin sensitivity check index, *WBC* white blood cell, *NEU* Neutrophil, *PLT* Platelet, *ALB* Albumin, *PA* Prealbumin, *CREA* Creatinine; TRIG Triglycerides, *CHOL* Total cholesterol, *HDL-C* High density liptein cholesterol, *LDL-C* Low-density lipoprotein cholesterol, IL-6 Interleukin-6, *25(OH) D* 25 hydroxy-vitamin D, *CRP* C-reactive protein, *NFS* Non-alcoholic fatty liver disease fibrosis score. Measurement data were expressed as median (interquartile range)

### IL-38 levels in NAFLD patients

**Fig. 1:**
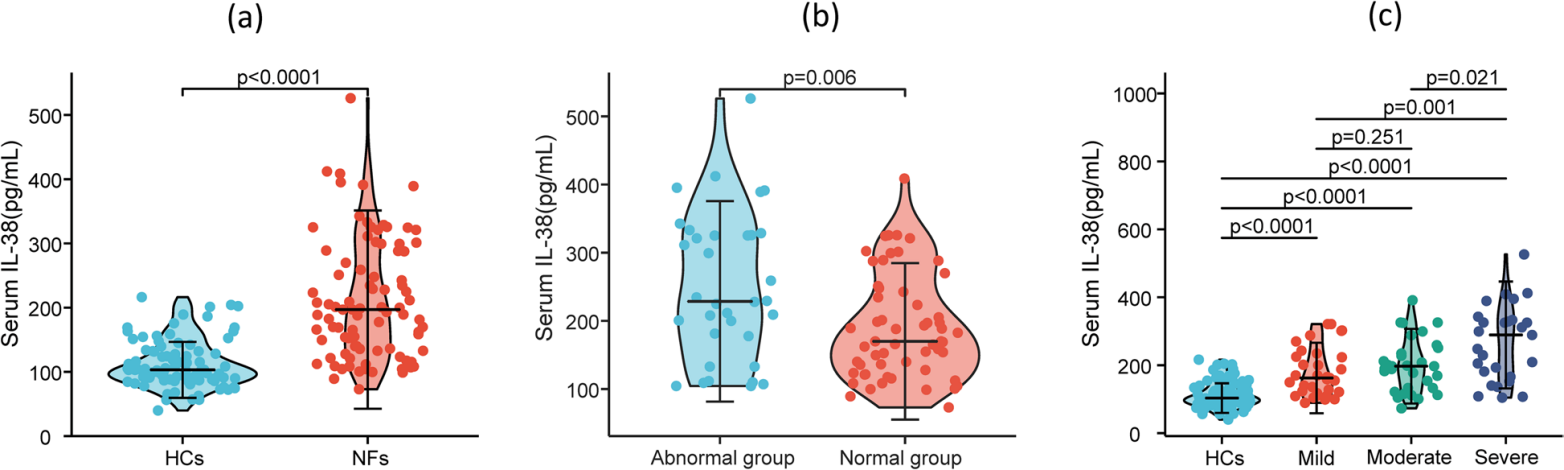
The level of serum IL-38 in NAFLD patients. **a** Comparison of serum IL-38 level in NAFLD cases and healthy control. **b** Comparison of serum IL-38 level in NAFLD patients with different ultrasonographic degrees. **c** Comparison of serum IL-38 level in NAFLD patients with abnormal liver enzymes or not

To characterise the status of IL-38 in NFALD cases, we detected the level of serum IL-38 using ELISA. Quantification analysis revealed circulating IL-38 in NFs was dramatically higher than HCs (*p* < 0.001, Fig. 1a). Based on the ultrasonographic degrees, NFs patients were categorized into three groups: mild group (*n* = 33), moderate group (*n* = 31), and severe group (*n* = 27). We further performed subgroup analysis within these grading groups, and the results showed that cases in the severe group had the highest levels of IL-38, followed by the moderate and mild groups (Fig. 1b). Furthermore, the IL-38 levels of patients with abnormal liver enzyme levels (ALT and/or AST) were higher than in those with normal liver enzyme levels (Fig. 1c). This result suggested that circulating IL-38 might involve in NAFLD pathogenesis.

### Characteristics of participants according to serum IL-38 tertiles

Participants were classified into tertiles (low, medium, or high) based on serum IL-38 levels to further investigate the correlation these and NAFLD. Table 2 displayed the baseline characteristics of all subjects. Compared with individuals in the lower tertile of IL-38 (T1), those expressing higher levels of IL-38 had higher ALT, AST, FBG, HOMA-IR, CRP, INS, CpS, WBC, NEU, PA, TRIG, and BMI, but lower baseline levels of QUICK, HDL-C, and 25(OH)D. Furthermore, with increasing IL-38, the proportion of NAFLD patients in T1, T2, and T3 was 13.33, 48.39, and 88.33%, respectively, exhibiting a clear increasing trend (*p* < 0.0001 for the trend).

**Table 2:** Clinical and laboratory characteristics by tertile distribution of serum IL-38 level

Indexes	T1	T2	T3	Statistical value, p	p1,p2,p3
Age	32.00(24.00,41.25)	35.50(30.00,42.00)	32.50(25.75,39.00)	4.424,0.110	0.176,1.000,0.250
ALT	16.35(10.58,21.33)	19.80(13.93,31.28)	31.00(19.15,51.48)	*30.437,< 0.0001*	*0.027,< 0.0001,0.010*
AST	20.60(18.28,24.20)	23.95(18.68,30.98)	30.85(23.63,41.83)	*27.725,< 0.0001*	0.114,*< 0.0001,0.004*
PLT	233.50(194.5286.75)	236.00(204.25,272.75)	220.50(181.00,255.00)	*6.337,0.042*	1.000,0.080,0.095
NFS	−3.40(−4.02,-2.65)	− 3.01(− 3.72,-2.50)	−2.86(− 3.51,-2.28)	*7.982,0.018*	0.272,*0.015*,0.756
FBG	4.65(4.44,4.89)	4.81(4.61,5.32)	4.92(4.64,5.48)	*13.605,0.001*	*0.010,0.002,*1.000
HOMA-IR	1.40(1.07,1.65)	1.88(1.43,2.50)	2.78(1.92,4.14)	*50.878,< 0.0001*	*0.001,< 0.0001,0.002*
QUICK	0.67(0.64,0.72)	0.615(0.57,0.66)	0.56(0.51,0.61)	*50.878,< 0.0001*	*0.001,< 0.0001,0.002*
CRP	0.16(0.13,0.23)	0.22(0.16,0.38)	0.23(0.15,0.35)	*10.633,0.005*	*0.010,0.020*,1.000
INS (mU/L)	6.68(5.36,7.68)	8.47(6.68,10.97)	12.69(9.21,15.63)	*46.278,< 0.0001*	*0.004,< 0.0001,0.001*
CpS	1.74(1.49,2.05)	2.22(1.83,2.75)	2.705(2.43,3.46)	*46.060,< 0.0001*	*0.001,< 0.0001,0.003*
HbA1c	5.30(5.18,5.53)	5.45(5.23,5.80)	5.60(5.30,5.90)	*12.448,0.002*	0.064,*0.002*,0.694
WBC	5.37(4.565,5.91)	6.33(5.10,7.58)	6.11(5.50,7.42)	*20.186,< 0.0001*	*0.001,< 0.0001*,1.000
NEU	2.93(2.34,3.56)	3.59(2.79,4.74)	3.44(2.78,4.30)	*15.887,< 0.0001*	*0.001,0.006*,1.000
ALB	49.55(47.68,51.23)	48.85(47.50,50.28)	49.00(47.83,51.15)	2.258,0.323	0.432,1.000,0.918
PA	247.50(222.50,271.50)	259.50(229.00,286.00)	285.00(267.00,317.00)	*22.438,< 0.0001*	0.552,*< 0.0001,0.003*
Urea	4.57(4.17,5.55)	4.41(3.79,5.25)	4.77(3.86,5.52)	1.825,0.401	0.668,1.000,0.806
CREA	59.00(52.75,70.25)	63.50(52.25,74.00)	71.50(62.50,78.00)	*13.852,0.001*	0.924,*0.001*,*0.027*
TRIG	0.99(0.73,1.20)	1.42(0.90,2.23)	1.96(1.41,2.98)	*53.150,< 0.0001*	*< 0.0001,< 0.0001,0.005*
CHOL	4.69(4.13,5.19)	4.67(4.11,5.44)	4.99(4.67,5.63)	*7.556,0.023*	1.000,*0.028*,0.112
HDL-C	1.54(1.36,1.76)	1.37(1.14,1.62)	1.27(1.07,1.46)	*27.448,< 0.0001*	*0.002,< 0.0001*,0.219
LDL-C	2.66(2.31,2.90)	2.63(2.25,3.33)	2.89(2.48,3.19)	5.880,0.053	1.000,0.053,0.306
BMI	21.78(19.91,24.02)	24.23 (21.32,26.26)	25.01 (23.65,27.32)	*30.499,< 0.0001*	*0.002,< 0.0001*,0.091
IL-6	1.60(0.98,2.48)	1.98(1.13,2.79)	2.43(1.55,3.40)	*12.447,0.002*	0.504,*0.001*,0.095
25(OH)D	19.80(17.20,22.65)	18.85(15.80,22.25)	15.75(12.43,19.83)	*18.564,< 0.0001*	0.769,*< 0.0001,0.007*
NF (%)	13.33	48.39	88.33	*67.600,0000*	*< 0.0001,< 0.0001,< 0.0001*

*p1, T1* vs *T2; p2, T1* vs *T3; p3, T2* vs *T3. BMI* Body mass index, *ALT* Alanine aminotransferase, *AST* Aspartate aminotransferase, *FBG* Fasting blood glucose, *INS* Insulin, *CpS* C-peptide, *HbA1c* Hemoglobin A1c, *HOMA-IR* Homoeostasis model assessment of IR, *QUICK* Quantitative insulin sensitivity check index, *WBC* White blood cell, *NEU* Neutrophil, *PLT* Platelet, *ALB* Albumin, *PA* Prealbumin, *CREA* Creatinine, *TRIG* Triglycerides, *CHOL* Total cholesterol, *HDL-C* High density liptein cholesterol, *LDL-C* Low-density lipoprotein cholesterol, *IL-6* Interleukin-6, *25(OH) D* 25 hydroxy-vitamin D, *CRP* C-reactive protein, *NFS* Non-alcoholic fatty liver disease fibrosis score. Measurement data were expressed as median (interquartile range)

### Serum IL-38 and anthropometric/biochemical parameters correlations in NAFLD patients

**Fig. 2:**
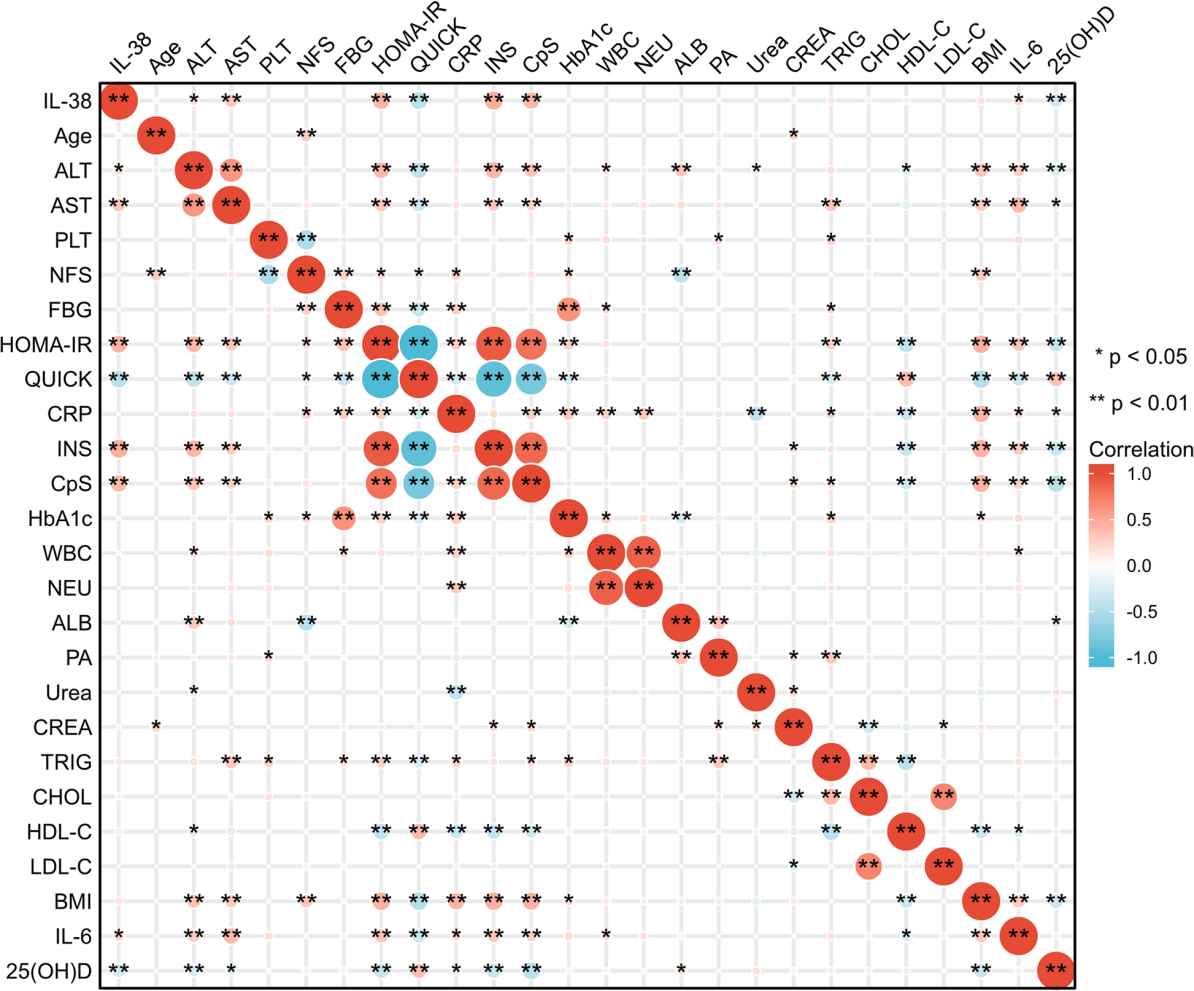
Correlation analyses between serum IL-38 levels with liver status, glucose metabolism and inflammation-related markers

In NFs, the correlations between serum IL-38 and demographic or anthropometric parameters (age and BMI); liver enzymes (ALT and AST); lipids (CHOL, HDL-C, LDL-C, and TRIG); glucose metabolism (FBG, HbA1c, INS, CpS, HOMA-IR, and QUICK); inflammation indices (WBC, NEU, CRP, and IL-6); indicators of kidney function (urea and CREA); and 25(OH) D were further explored (Fig. 2). Significant positive associations of serum IL-38 levels with HOMA-IR, INS, and CpS were found, whereas QUICK was negatively associated with IL-38 level in glucose metabolism. Positive associations were observed between IL-38 and ALT or AST levels. When inflammation parameters were evaluated, a positive association between serum IL-38 and IL-6 was also observed. IL-38 and 25(OH) D were found to have a negative correlation.

### Odd ratios (ORs) of NAFLD by IL-38 tertiles

The association between serum IL-38 and ORs of NAFLD was evaluated with a multivariable logistic regression analysis. The ORs for NAFLD in T2 or T3 vs. T1 for serum IL-38 were shown in Table 3. The OR of T2 vs. T1 was statistically significant after adjusting age, sex, BMI, ALT, and AST, but it was not significant after adjustment for HDL-C and LDL-C. In the multivariate analysis with adjustment for all the above explanatory variables, the OR of T3 vs. T1 remained a significantly positive determinant of NAFLD. These results indicated that higher serum IL-38 was linked to a higher risk of NAFLD.

**Table 3:** The association of serum IL-38 levels with risk of NAFLD in different models

Comparisons	Models	Odds ratios (95% CI)	***p*** value
T1 vs T2	M 1	6.094 (2.488–14.923)	< 0.0001*
	M 2	5.944 (2.394–14.758)	< 0.0001*
	M 3	3.677 (1.269–10.648)	0.016*
	M 4	3.357 (1.075–10.485)	0.037*
	M 5	3.450 (0.975–12.199)	0.055
T1 vs T3	M 1	49.214(16.644–145.519)	< 0.0001*
	M 2	53.999(17.480–166.757)	< 0.0001*
	M 3	73.028(13.565–393.164)	< 0.0001*
	M 4	51.614(8.920–298.660)	< 0.0001*
	M 5	40.104(5.905–272.393)	< 0.0001*

M 1: unadjusted, M 2: adjusted age and gender, M 3: adjusted age, gender and BMI, M 4: adjusted age, gender, BMI, ALT and AST, M 5: adjusted age, gender, BMI, ALT, AST, HDL-C and LDL-C

### ROC curve of IL-38

**Fig. 3:**
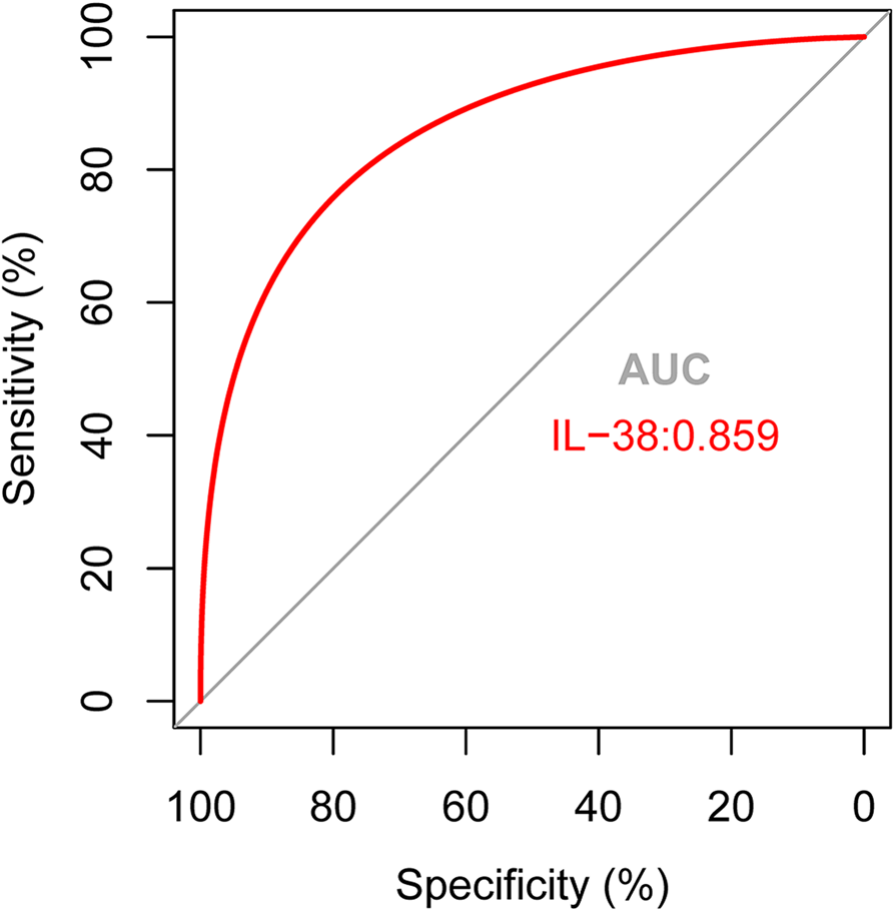
ROC curve of serum IL-38 levels in diagnosing NAFLD

The ROC curve of serum IL-38 was plotted to predict NAFLD (Fig. 3.). The area under the ROC curve (AUC) was 0.859 (95% CI, 0.807–0.911, *p* < 0.0001). And at the cut-off value of 132.53 pg/mL for serum IL-38, the sensitivity and specificity were 79.12 and 74.73%, severally.

### Variables related to serum IL-38

In all subjects, Spearman’s analysis revealed that serum IL-38 level was correlated with ALT, AST, PLT, FBG, HOMA-IR, QUICK, INS, CpS, HbA1c, WBC, PA, CREA, TRIG, HDL-C, BMI, IL-6, and 25(OH)D. Based on these results, we performed linear regression analysis using serum IL-38 level as the dependent variable and these above indexes as independent variables. AST, HOMA-IR, and TRIG were all independently linked to serum IL-38 in a multivariate linear regression model (Table 4).

**Table 4:** Multivariate linear regression analysis of variables associated with serum IL-38

	β	t	***p*** value
ALT	−0.032	−0.328	0.743
AST	0.230	2.229	0.027*
PLT	−0.069	−1.16	0.248
FBG	−0.277	−1.51	0.133
HOMA-IR	0.839	2.506	0.013*
QUICK	−0.073	−0.563	*0.574*
INS	−0.323	−1.002	0.318
CpS	−0.098	−0.739	0.461
HbA1c	−0.014	−0.11	0.913
WBC	0.043	0.722	0.471
PA	0.082	1.169	0.244
CREA	0.016	0.23	0.818
TRIG	0.191	2.557	0.011*
HDL-C	−0.005	−0.069	0.945
BMI	0.068	0.823	0.412
IL-6	0.049	0.801	0.424
25(OH)D	−0.065	−1.001	0.318

*ALT* Alanine aminotransferase, *AST* Aspartate aminotransferase, *PLT* Platelet, *FBG* Fasting blood glucose, *HOMA-IR* Homoeostasis model assessment of IR, *QUICK* Quantitative insulin sensitivity check index, *INS* Insulin, *CpS* C-peptide, *HbA1c* Hemoglobin A1c, *WBC* White blood cell, *PA* Prealbumin, *CREA* Creatinine, *TRIG* Triglycerides, *HDL-C* High density liptein cholesterol, *BMI* Body mass index, *IL-6* Interleukin-6, *25(OH) D* 25 hydroxy-vitamin D

## Discussion

The current study demonstrated that serum IL-38 was considerably upregulated in NAFLD patients. Simultaneously, the frequency of NAFLD increased as serum IL-38 levels increased, as revealed by the analysis of serum IL-38 level tertiles. Furthermore, level of IL-38 was related with NAFLD severity, as measured by ultrasonography. Finally, correlation analyses revealed significant relationships between serum IL-38 levels and parameters related to liver injury, inflammation, and insulin resistance in individuals with NAFLD or in all participants. Indicators of liver injury, inflammation, lipids and insulin resistance were considerably higher in NAFLD cases than in controls. As we know, this is the first study to exhibit a link between serum IL-38 and NAFLD.

Investigations have showed that IL-38 participated in pathogenic processes of several inflammatory disorders [22–24]. Understanding IL-38 and its biological roles may bring about the exploration of new diagnostic and therapeutic strategies. A previous study on ischaemic stroke revealed that serum IL-38 levels might be an early predictor for this condition [25]. In a study of systemic lupus erythematosus, high level of circulating IL-38 was found to be correlated with high risk of renal complications [26]. Moreover, in osteoarthritis patients, higher IL-38 levels indicated severe disease activity [27]. In Graves’ disease and Hashimoto’s thyroiditis, two types of thyroid inflammatory diseases, low serum IL-38 levels were observed [28]. These results suggest that IL-38 may exhibit anti- or pro-inflammatory actions in different pathological states, similar to its family members [15]. Specifically, in metabolic diseases, researchers have found that plasma IL-38 increased in diabetes patients and was positively associated with markers of liver and kidney function, glucose metabolism, and serum lipids, which supported IL-38 as an associated factor of inflammation and/or altered metabolism [15, 19]. Recent studies have established associations between NAFLD, inflammatory responses, and insulin resistance [29–32]. IL-38 has anti-inflammatory effects, and it was found that IL-38 can increase the expression of GATA3 and glucose transporter type 4 mRNA in adipocytes and inhibit the production of IL-6 and IL-1β, suggesting that IL-38 can inhibit human adipocyte differentiation and inflammatory response [33]. The hydrodynamic delivery of recombinant IL-38 lowered liver fat content and the degree of insulin resistance in obese mice [18]. In addition, it attenuated the secretion of inflammatory mediators. NAFLD is charactered by abnormal liver fat deposition and insulin resistance, both of which have been proven to be closely linked to inflammation [34]. As observed in the present study, indicators related to these pathological processes exhibited abnormalities. Accordingly, we hypothesise that IL-38 may be related to the homeostasis of liver metabolism in NAFLD.

Our study revealed a higher concentration of anti-inflammatory IL-38 in NAFLD subjects, suggesting IL-38 could be part of a feedback loop to attenuate the observed inflammation. However, as the level of IL-38 upregulated in the tertiles, the frequency and OR of NAFLD also increased. Furthermore, serum levels of IL-38 showed a positive correlation with NAFLD when analysed using binary logistic regression and the ROC curve. Lastly, higher IL-38 levels were observed to be closely correlated with insulin resistance (INS, CpS, HOMA-IR, and QUICK), liver injury (AST and ALT), and inflammation [IL-6 and 25(OH)D]. Furthermore, the results also demonstrated AST, HOMA-IR, and TRIG were independently associated with IL-38 levels in all participants. Indeed, increased serum IL-38 has been found in chronic hepatitis B sufferers, indicating ongoing liver damage, as reflected by its positive correlation with IL-6 and/or AST [35]. Moreover, in a type 2 diabetes case-control research, IL-38 was also reported to be relevant for HOMA-IR and TRIG [19]. For the correlation between IL-38 and 25(OH) D, reports are scarce; however, NAFLD has been associated with reduced levels of 25(OH)D [36], which was confirmed to contribute to oxidative stress modulation, secretion of cytokines, and hepatocyte apoptosis [37–39]. These findings suggest that the level of IL-38 could be a measure of liver injury and/or insulin resistance; however, we were unable to determine whether a higher level of IL-38 was a provoker of insulin resistance or liver injury. Nevertheless, our results support the idea that IL-38 participates in NAFLD pathogenesis, possibly by modulating inflammation and insulin resistance. The identification of serum biomarkers associated with NAFLD is of clinical importance [40]. We investigated whether serum IL-38 level could be utilized as a biomarker of NAFLD. The ROC curve displayed a statistically significant AUC of 0.859. However, due to the relatively small sample size included in this study, the adequacy of serum IL-38 to distinguish NAFLD needs to be further approved in studies with large sample populations. Other limitations of the present research include the fact that all participants enrolled were from an urban area in China, which may have led to selection bias. Additionally, the gold standard for NAFLD diagnosis is liver biopsy instead of ultrasonography, the method selected for the present study. Moreover, on account of the nature of this investigation, we cannot build a causal link between serum IL-38 levels and NAFLD.

## Conclusions

Circulating IL-38 concentration was associated with NAFLD. Furthermore, increased IL-38 was also accompanied by a higher NAFLD risk and correlated with indicators of insulin sensitivity and liver injury. This suggests that IL-38 could serve as a promising biomarker in NAFLD. However, further in-depth exploration is required to illuminate the mechanisms behind the link between IL-38 and NAFLD.

## Data Availability

The data of the current study are available from the corresponding author on reasonable request.

## Abbreviations

NAFLD: Non-alcoholic fatty liver disease
NFs: NAFLD patients
HCs: Healthy controls
ALT: Alanine aminotransferase
AST: Aspartate aminotransferase
ALB: Albumin
PA: Prealbumin
TRIG: Triglycerides
HDL-C: High density liptein cholesterol
CREA: Creatinine
CHOL: Total cholesterol
LDL-C: Low-density lipoprotein cholesterol
INS: Insulin
CpS: C-peptide
IL-6: Interleukin-6
FBG: Fasting blood glucose
HbA1c: Hemoglobin A1c
CRP: C-reactive protein
25(OH)D: 25 hydroxy-vitamin D
BMI: Body mass index
NFS: Non-alcoholic fatty liver disease fibrosis score
HOMA-IR: Homoeostasis model assessment of IR
QUICK: Quantitative insulin sensitivity check index
WBC: White blood cell
NEU: Neutrophil
PLT: Platelet
ELISA: Enzyme-linked immunosorbent assay
ROC: Receiver operating characteristic
ORs: Odd ratios
